# The dilemma for lipid productivity in green microalgae: importance of substrate provision in improving oil yield without sacrificing growth

**DOI:** 10.1186/s13068-016-0671-2

**Published:** 2016-11-22

**Authors:** Kenneth Wei Min Tan, Yuan Kun Lee

**Affiliations:** Department of Microbiology and Immunology, Yong Loo Lin School of Medicine, National University of Singapore, Singapore, 117545 Singapore

**Keywords:** Microalgae, Lipid productivity, Fatty acid, Nitrogen depletion, Acetyl-CoA, NADPH, ATP:citrate lyase, Malic enzyme, Glucose-6-phosphate dehydrogenase, Pyruvate dehydrogenase

## Abstract

Rising oil prices and concerns over climate change have resulted in more emphasis on research into renewable biofuels from microalgae. Unlike plants, green microalgae have higher biomass productivity, will not compete with food and agriculture, and do not require fertile land for cultivation. However, microalgae biofuels currently suffer from high capital and operating costs due to low yields and costly extraction methods. Microalgae grown under optimal conditions produce large amounts of biomass but with low neutral lipid content, while microalgae grown in nutrient starvation accumulate high levels of neutral lipids but are slow growing. Producing lipids while maintaining high growth rates is vital for biofuel production because high biomass productivity increases yield per harvest volume while high lipid content decreases the cost of extraction per unit product. Therefore, there is a need for metabolic engineering of microalgae to constitutively produce high amounts of lipids without sacrificing growth. Substrate availability is a rate-limiting step in balancing growth and fatty acid (FA) production because both biomass and FA synthesis pathways compete for the same substrates, namely acetyl-CoA and NADPH. In this review, we discuss the efforts made for improving biofuel production in plants and microorganisms, the challenges faced in achieving lipid productivity, and the important role of precursor supply for FA synthesis. The main focus is placed on the enzymes which catalyzed the reactions supplying acetyl-CoA and NADPH.

## Background

Diminishing fossil fuel reserves, rising oil prices, and concerns over climate change due to increasing atmospheric CO_2_ levels have renewed support for alternative and renewable energy sources [1]. In particular, biofuels derived from photosynthetic microorganisms such as cyanobacteria and microalgae have received considerable interest because compared to plants, they require smaller land area that does not need to be arable and can be cultivated in saltwater systems which will not directly compete with resources necessary for agricultural food production [2]. Furthermore, microalgae are efficient in converting solar energy and sequestering CO_2_ into storage lipids, with several species having higher biomass production rates and containing a higher percentage of oil than terrestrial plants [3, 4]. Even by conservative standards, microalgae are still predicted to produce 10 times more biodiesel per unit area of land compared to terrestrial oleaginous crops [5]. However, microalgal biofuel production based on present yields and extraction methods is unlikely to be economically viable as algae fuel currently cost more per unit mass due to the high capital and operating costs [6]. Although the U.S. Department of Energy’s Aquatic Species Program identified around 300 species of microalgae that are desirable for biofuel production [7], continued engineering of microalgae is still required to unlock the feasibility of algal strains to serve as a factory for the production of cost-efficient biofuels.

Microalgae utilize solar energy, water, and CO_2_ which are assimilated into the reserve storage components of carbohydrates, lipids, and proteins. Of these three biochemical forms, lipids have the highest energy content [8] which make them favorable high-value molecules. Harvested lipids typically undergo transesterification with methanol to be converted into fatty acid methyl esters that can be used as a transportation fuel in the form of biodiesel [9]. The remaining biomass may be converted into methane gas or the carbohydrates may be transformed into ethanol by dark fermentation [10, 11]. Although microalgae generally use starch as their primary carbon storage compound, some strains accumulate neutral lipids, mainly in the form of triacylglycerols (TAG) under environmental stress conditions such as nitrogen limitation [12]. During logarithmic growth, microalgae do not produce large amounts of storage lipids. However, in response to environmental stress they reduce their proliferation and start producing TAG [13]. The accumulation of TAG likely occurs as a means of creating an energy deposit that can be readily utilized in response to a more favorable environment allowing for rapid growth [14]. While an increase in TAG production during nitrogen deprivation is ideal, reduced growth caused by nutrient deficiency hampers the use of this strategy for producing biofuel as the decrease in biomass productivity would reduce overall yield [15]. Therefore, a new focus in microalgal biofuel research is to genetically engineer an ideal strain which will have high growth rate while possessing high lipid content.

In this review, we will discuss potential bottlenecks deterring the economic application of microalgae biofuels, and the possible ways to overcome them. The importance of productivity will be highlighted, along with a review of genetic modifications that may increase lipid accumulation without compromising growth. Particular emphasis is placed on translating the various approaches into microalgae as the source for biofuel production. For the purpose of this review, we are focusing the discussion towards its applications in green algae (Chlorophyta division) and *Nannochloropsis,* as these species are frequently studied for the production of renewable biofuels.

### Strategies targeting the overproduction of lipids

To date, most studies seeking to improve TAG accumulation in photosynthetic organisms focus on the fatty acid (FA) synthesis pathway, which provides the acyl-coenzyme A (CoA) substrates for TAG synthesis. Overexpression of the enzyme acetyl-CoA carboxylase (ACCase), believed to catalyze the important rate-limiting step in FA synthesis, has been performed in diatoms [16] and plants [17, 18] but only minor (5%), if any, increase in lipid content was observed. Increasing the expression of another enzyme in FA synthesis, 3-ketoacyl-acyl carrier protein synthase (KAS) III, was also not successful in improving the lipid content in three species of plants [19]. On the other hand, manipulation of genes involved in TAG assembly has achieved better results. One of the more successful attempts is the overexpression of glycerol-3-phosphate dehydrogenase (G3PDH), an enzyme involved in supplying glycerol-3-phosphate (G3P) required for TAG formation. By overexpressing G3PDH in the developing seeds of *Brassica napus*, Vigeolas and colleagues [20] observed a three- to four-fold increase in G3P and a 40% increase in final seed oil content, suggesting that the genes involved in the substrate synthesis are also important for increasing lipid production. The overexpression of diacylglycerol acyltransferase (DGAT), the major enzyme catalyzing the final step of TAG synthesis, in the seeds of *Arabidopsis thaliana* [21] and soybean [22] also resulted in a substantial increase in the oil content. Despite the success in plants, overexpression of the three DGAT homologue genes identified in *Chlamydomonas reinhardtii* did not boost intracellular TAG during standard growth or nitrogen-depleted conditions [23]. Transcriptomic analysis of *C. reinhardtii* subjected to nitrogen depletion showed low levels of DGAT consistently [24], while the oleaginous microalga *Neochloris oleoabundans* also presented no changes in the DGAT expression between normal growth and nitrogen-limited cultures [25]. This suggests that increasing DGAT expression may not be effective in increasing lipid content for microalgae. One possibility is that TAG assembly might differ between plants and microalgae [26], such as the location of TAG synthesis. In plants, the synthesis of TAG occurs at the ER, but in microalgae a large proportion of TAG is synthesized at the plastids in parallel to FA synthesis [27, 28]. This would affect the selection of genes because overexpression of ER-localized enzymes may not have much effect in increasing TAG content. However, all known *Chlamydomonas* DGATs lack plastid targeting sequences, highlighting the possibility that novel enzymes might be involved in the assembly of TAG in *Chlamydomonas* plastids [26]. Researchers have found plastidial isoforms of G3PDH in *Dunaliella* [29–31] and recently in *C. reinhardtii* [32] which may suggest a specific chloroplast pathway for TAG synthesis in microalgae.

While little information is available regarding the metabolic pathways in microalgae, the *de novo* FA synthesis pathway is consistent with those proposed for higher plants [25, 33]. FA elongation by long-chain KAS enzymes ends with the production of 16–18C fatty acyl groups esterified to acyl carrier protein (ACP), which are used by the cell to synthesize membrane lipids [34]. The buildup of fatty acyl-ACPs can regulate the rate of FA synthesis by feedback inhibition of ACCase [35] and KAS [36]. However, acyl-ACP thioesterases (TEs) can reduce this inhibition by hydrolyzing the acyl-ACP into free FAs, which are converted into acyl-CoA and released from the chloroplast to be incorporated into TAGs [34]. The expressions of endogenous TEs such as oleoyl-ACP hydrolase and acyl-ACP thioesterase A were found to be upregulated in microalgae subjected to nitrogen-limiting conditions [24, 25]. In addition, the expression of TE showed a linear relationship with FA synthesis in *Haematococcus pluvialis*, indicating that TE could be involved in a key rate-limiting step for FA synthesis [37]. Based on this principle, increasing the activity of TEs appears to be a good strategy to promote the continuous production of FAs and channeling them to storage lipids rather than membrane lipids. Indeed, the overexpression of TEs has been shown to increase total FAs in *E. coli* [38] and cyanobacteria [39], and in microalgae by up to 72% [40]. As different TEs are specific for different FA chain lengths, manipulating the type of TE could also result in overproduction of short- and medium-chain FAs which are preferred for biodiesel production. For example, overproducing a TE from *Umbellularia californica* in *E. coli* and cyanobacteria increased the accumulation of myristate (14:0) and laurate (12:0) [39, 41], which are beneficial as biofuels because saturated carbon chains have higher octane rating and are more resistant to oxidation during storage. Furthermore, targeted engineering of TEs with KAS enzymes specific for short- or medium-chain length FAs would enable the synthesis of customized biofuel molecules that are industrially relevant [42, 43].

Another possible approach to complement an increase in lipid content is decreasing lipid catabolism. Genes involved in β-oxidation of FAs, as well as the activation of free FAs could be targeted for deletion. Knocking out the acyl-CoA synthetase (ACSL) gene, which targets free FAs for β-oxidation in *Escherichia coli* [38] and *Saccharomyces cerevisiae* [44, 45] resulted in a significant increase in free FA production; knockout of ACSL in *S*. *cerevisiae* also led to extracellular FA secretion [44, 46]. In addition, ACSL is also downregulated in *N. oleoabundans* during nitrogen-limited conditions when lipids are accumulated [25], suggesting that this enzyme could be a major factor in controlling the flux of FAs toward their degradation by β-oxidation. Enzymes of β-oxidation in microalgae include acyl-CoA dehydrogenase (ACD) and acyl-CoA oxidase (ACOX) which are responsible for the breakdown of FAs in the mitochondria and peroxisomes, respectively, and could be promising targets for microRNA silencing. Transcript levels of ACOX decreased in *C. reinhardtii* under nitrogen depletion [24], but ACD levels were upregulated in N-depleted *Nannochloropsis* [47], indicating the varied roles these pathways might play in lipid accumulation. On one hand, inhibiting β-oxidation would prevent the loss of lipids and result in intracellular buildup of acyl-CoAs, as observed in *A. thaliana* mutants lacking ACOX [48]. On the other hand, an increase in β-oxidation could provide for enhanced recycling of carbon skeletons from degraded membrane lipids for TAG synthesis.

The importance of lipid degradation and turnover was recently demonstrated in *A. thaliana* leaves by Fan et al. [126]. The study found that while overexpression of phospholipid:diacylglycerol acyltransferase (PDAT) did not substantially increase TAGs relative to wild type, concurrent disruption of a TAG lipase greatly increased TAG content by up to 14-fold, suggesting that preventing TAG degradation could play a bigger role in lipid accumulation. However, membrane lipid composition was altered in the double mutant, leading to compromised growth and development. Their results indicate that TAG lipases are key regulators of FA turnover by degrading TAGs and redirecting acyl-CoAs for membrane lipid synthesis, thereby maintaining lipid homeostasis. The importance of TAG lipases is made more pronounced when the overexpression of PDAT channels phospholipids away from membrane lipids. In contrast, inhibiting TAG degradation alone without interfering with membrane lipid composition could prove more useful. A recent study by Trentacoste et al. found that knocking down a multifunctional lipase/phospholipase/acyltransferase increases the lipid content without compromising growth in the diatom *Thalassiosira pseudonana* [49]. During exponential growth, the mutants showed a 2.4- to 3.3-fold increase in the lipid content relative to the wild type, and an even higher fourfold increase during silicon starvation. As lipases specifically target the release of FAs from TAG and lipid droplets, the authors propose that reducing lipid catabolism would have minimal impact on growth compared to strategies which disrupt carbohydrate pools. For instance, Li et al. had previously disrupted the ADP-glucose pyrophosphorylase gene involved in starch synthesis in *C. reinhardtii* [50]. While the microalgae accumulated up to an eightfold increase in TAGs during nitrogen deprivation, it also resulted in reduced photosynthetic efficiency and growth impairment. Consequently, it was discovered that a major shift in carbon flux from starch to lipid synthesis might compromise cellular growth and biomass productivity as polysaccharides are consumed to support cell division [14, 51–53]. Hence, manipulation of genes involved in starch synthesis pathways might not be a practical approach due to the shift in balance between lipid accumulation and growth; increasing the efficiency of one pathway without accounting for the other will likely sacrifice overall yield.

### Overproduction without sacrificing growth and productivity

The extent of lipid accumulation in microalgae is influenced by stress factors such as nutrient levels and culture conditions. Under optimal growth conditions, large amounts of algal biomass are produced, but with relatively low lipid content. In nutrient stress conditions, cells have high lipid contents but are typically slow growing. Lipid content alone does not allow for rational species selection as faster growing species may demonstrate lipid productivity greater than those with high lipid content [54]. Producing lipids while also maintaining high growth rates and increasing biomass is therefore vital for algal biofuel production on large economic scales. High biomass productivity increases yield per harvest volume while high lipid content decreases the cost of extraction per unit product [54]. Rapid growth rate also provides a competitive advantage over contaminating algal species in outdoor cultures, and requires less culture space due to a higher cell density per area. Thus, cost-effective production of lipids for commercial use ideally requires deregulated microalgae which constitutively accumulate high amounts of lipids, regardless of environmental conditions that impede growth.

Green algae have proven to be recalcitrant to oil accumulation as their metabolism favors starch accumulation [55, 56]. However, under stressed conditions they begin to accumulate higher amounts of TAGs, akin to plant oil seeds [56]. This suggests that oleaginicity could be initiated by the diversion of carbon flux from central carbon metabolism towards lipids instead of starch. For example, although the oleaginous green algae *Pseudochlorococcum sp.* uses starch as a primary storage product, it was found to accumulate up to 52% lipids under nitrogen depletion because it is able to convert starch into lipids [57]. Inhibition of starch synthesis in microalgae may therefore not only reduce growth but also result in decreased lipid content. On the other hand, microalgae species that are capable of heterotrophic growth on sugars were able to enhance lipid accumulation by 900% compared with photoautotrophically grown cells [58]. Green algae such as *Chlorella zofingiensis* were able to achieve as much as 79.5% total lipids when fed with glucose and grown in darkness [58], compared to 65% lipids when it is grown photoautotrophically and subjected to nitrogen deprivation [59]. The difference in potential for lipid accumulation between these cells suggests that growth conditions and type of carbon source may play a defining role in the carbon flux towards storage of lipids. Taken together, these observations serve to highlight that alterations in central carbon metabolism could influence carbon partitioning towards oil accumulation in microalgae [56]. For biofuel applications, rather than inhibiting starch synthesis which is detrimental to growth, it might be useful to manipulate the flow of photosynthetic carbon towards the accumulation of lipids—rather than starch—in high biomass-producing microalgae species [54, 60].

Overexpression of genes that increase TAG synthesis alone is likely to reduce microalgae growth rate because TAG synthesis requires the supply of two substrates, acyl-CoA and G3P. G3P is derived from dihydroxyacetone phosphate (DHAP) produced from the Calvin cycle and glycolysis. Channeling of carbon away from either of these pathways to TAG synthesis would reduce the carbon available for pathways supporting cellular growth, such as the tricarboxylic acid (TCA) cycle. Scientists have overexpressed G3PDH in the diatom *Phaeodactylum tricornutum* leading to a 60% increase in neutral lipids during stationary phase, as a 6.8-fold increase in glycerol content provided the backbone for TAG synthesis [61]. However, it also resulted in a 20% decrease in cell growth, as overexpressing G3PDH promoted the conversion of DHAP to G3P, shunting carbon away from glycolysis and the TCA cycle to form glycerol. Likewise, overexpression of FA synthesis genes such as ACCase could result in reduced growth as the carbon required for acyl-CoA formation is tapped from acetyl-CoA, the key two-carbon metabolite shared between the TCA cycle and FA synthesis. As both of these pathways compete for acetyl-CoA, it is not surprising that overexpression of genes involved in FA synthesis produced only modest increases in the lipid content [62]. These evidences highlight substrate availability as a critical bottleneck to lipid synthesis because biomass and lipid production essentially compete for photosynthetic assimilate. Clearly, the solution to balancing growth and lipid accumulation should lie upstream of lipid-producing pathways.

Increasing evidence suggest that the “pulling” of carbon from FA and TAG synthesis pathways might not be as crucial as the “pushing” of carbon into FA synthesis. For instance, recent transcriptomic studies on carbon fixation in *P. tricornutum* [63, 64] found that the buildup of precursors such as acetyl-CoA and NADPH may provide a more significant contribution to TAG accumulation than the activity of ACCase. Following nitrogen deprivation, genes involved in carbon fixation, TCA cycle, and glycolysis were enhanced, possibly providing the resources necessary for carbon flux towards neutral lipid synthesis [64]. In contrast, RNA-seq performed on nitrogen-deprived *C. reinhardtii* showed no change in the transcript levels of genes encoding for FA synthesis [24], suggesting that the increased lipid content associated with nitrogen deprivation might be influenced by factors outside the FA synthesis pathway. Transcriptomics performed on oil palm revealed that supply of pyruvate in the plastids, rather than acyl assembly into TAGs, was the major contributing factor responsible for its oleagenicity [65], as transcript levels of enzymes involved in plastidial carbon metabolism including phosphofructokinase, pyruvate kinase, and pyruvate dehydrogenase subunits were more than 50-fold higher compared to date palm, a closely related species that accumulate almost exclusively sugars rather than oil. Surprisingly, despite a 100-fold difference in flux to lipids, most enzymes involved in TAG synthesis were expressed at similar levels in oil palm and date palm [65]. Notably, transcriptomic comparisons between oleaginous and non-oleaginous microbes revealed no significant changes in genes encoding enzymes directly involved in FA synthesis [66]. Rather, the availability of precursors appears to be the key point of transcriptional regulation contributing to oleagenicity in oil-accumulating microbes. Therefore, increasing the availability of precursors for both primary metabolism and FA synthesis could be a viable approach to simultaneously increase the yield of biomass and lipids in microalgae.

### Provision of substrates: Acetyl-CoA and NADPH

When cerulenin, an inhibitor of de novo FA synthesis, was added to *C. reinhardtii* cells, lipid droplet formation was strongly inhibited, indicating that a significant portion of storage lipids (i.e., TAGs) is likely derived from de novo FA synthesis [67]. High rates of de novo FA synthesis require a continuous supply of acetyl-CoA and NADPH [68]. During nitrogen starvation, carbon precursors in the form of acetyl-CoA rapidly rise, preceding TAG accumulation [69]. Reactions that provide for these precursor molecules lie outside the FA synthesis pathway which is common to all microorganisms, implying that the difference between non-oleaginous and oleaginous microorganisms might be the latter’s ability to direct more substrates to FAs [70]. Despite possessing the same pathway to synthesize FAs, non-oleaginous microorganisms typically do not accumulate FAs but instead divert carbon into polysaccharides [69, 70]. In high TAG-accumulating microalgae such as *Chlorella desiccata*, the levels of acetyl-CoA far exceeded those seen in moderate TAG accumulators such as *D. tertiolecta* and *C. reinhardtii* [69], suggesting that the availability of carbon precursors may limit TAG accumulation in green microalgae. Metabolic flux analysis performed in *Chlorella protothecoides* showed that carbon flow from acetyl-CoA to FA pools increased from 58 to 109% of glucose uptake during nitrogen-limited growth [71], indicating that algal cells substantially reorganize their metabolism to divert more acetyl-CoA towards lipid production. Increased NADPH pool in a *Chlorella pyrenoidosa* mutant overexpressing an *A. thaliana* NADH kinase (AtNADK3) has also been associated with enhanced lipid content of up to 110% [72] (Table 1). Oleaginicity is hence attributed to the presence and activity of enzymes responsible for the supply of acetyl-CoA and NADPH to FA synthesis. Enzymes contributing to the intracellular pool of acetyl-CoA include acetyl-CoA synthetase (ACS), ATP:citrate lyase (ACL), and pyruvate dehydrogenase complex (PDC) while those that provide for NADPH include NADP-malic enzyme (ME) and glucose-6-phosphate dehydrogenase (G6PDH).

**Table 1:** Influence of genes supplying substrates for lipid synthesis in transgenic strains

Genes	Description	Host species	Method of intervention	Effects (relative to control)	Inferred role	Ref.
PDK	Pyruvate dehydrogenase kinase	*Phaeodactylum tricornutum*	Antisense knockdown	33–82% more neutral lipids in 2 mutants	PDK deactivates Pyruvate dehydrogenase complex (PDC). Knocking down PDK increases acetyl-CoA production from pyruvate via PDC	[101]
NADK3	*Arabidopsis thaliana* NAD(H) kinase (AtNADK3)	*Chlorella pyrenoidosa*	Gene overexpression	Total lipid content increased by 45.3–110.4%; NADPH content increased by 39.3–79.9%	Heterologous NADH kinase increases NADPH which drives reductive biosynthesis reactions such as FA synthesis	[72]
ACS	*E. coli* Acetyl-CoA synthetase (ACS)	*Schizochytrium sp.*	Gene overexpression	Total lipid content increased by 6.4–11.4%; Biomass increased by 24.3–29.9%	Heterologous ACS overexpression improved utilization of acetate as a carbon resource for growth and lipid synthesis	[78]
ACL	*Mus musculus* ATP:citrate lyase	*Yarrowia lipolytica*	Gene overexpression	Total lipid content increased by 50.6–215.1%; Citrate content decreased by 32%	Heterologous expression of ACL with a low Km value for citrate increases lipid synthesis by providing more cytosolic acetyl-CoA as substrates	[138]
PDH E1α	E1 alpha subunit of the Pyruvate Dehydrogenase Complex	*Chlamydomonas reinhardtii*	Artificial microRNA knockdown	Total lipid content decreased by 25–40%; Lower chlorophyll content, lower photosynthetic yield on PSII, and lower biomass in mutants	PDC serves an essential role in the supply of carbon precursors for FA synthesis under photoautotrophy	[100]
ME	Malic enzyme	*Phaeodactylum tricornutum*	Gene overexpression	2.3- to 2.5-fold more neutral lipids in 2 mutants; growth rate unaffected	ME could increase lipid synthesis without affecting biomass accumulation by providing NADPH	[128]
ME	*Phaeodactylum tricornutum* malic enzyme (PtME)	*Chlorella pyrenoidosa*	Gene overexpression	2.4- to 3.2-fold more neutral lipids in 2 mutants; growth rate unaffected	Heterologous ME could increase lipid synthesis without affecting biomass accumulation by providing NADPH	[129]
G6PDH	Glucose-6-phosphate dehydrogenase	*Mortierella alpina*	RNAi knockdown	Total lipid content decreased by 50%; NADPH content decreased by 40%	G6PDH is a critical component of the Oxidative Pentose Phosphate Pathway which enables efficient lipid synthesis	[66]
ACL/ME	ATP:citrate lyase and Malic enzyme	*Yarrowia lipolytica*	Five gene modifications including overexpression of ME and ACL subunit 1 and subunit 2	Total lipid content increased to 74%, a 15-fold improvement over wild type (16.8%)	ACL and ME cooperatively divert carbon precursors and reducing power towards lipid synthesis, and in conjunction with other modifications, lead to enhanced lipid accumulation	[85]

#### Acetyl-CoA synthetase

Acetyl-CoA is a central metabolite involved in various physiological pathways linked with anabolism and catabolism, such as FA synthesis and the TCA cycle. Besides being a precursor for biochemical reactions, acetyl-CoA also serves in post-translational modifications, namely acetylation, of proteins including histones and transcription factors [73]. Due to its versatility in functions, its synthesis is suggested to occur in different compartments under the control of various enzymes. While much of acetyl-CoA production still remains unknown in microalgae, mechanisms that include the three enzymes (ACS, PDC, and ACL) are thought to be involved [73]. ACS carries out the reversible reaction which converts acetate to acetyl-CoA, and was once thought to be the primary source of acetyl-CoA for FA synthesis as it was the first enzyme identified to produce acetyl-CoA in plastids [74]. However, subsequent studies have refuted that claim as its expression was not correlated with increased lipids [75] and mutants lacking ACS still fix CO_2_ into FAs at the same rate as wild type [76]. In addition, increased lipid production in microalgae appears to be dependent on exogenously supplied acetate [27, 77], suggesting that the cells themselves do not produce enough physiological acetate required by ACS for it to be a primary source of acetyl-CoA. An exception exists where heterotrophically grown *Schizochytrium* sp. supplemented with glucose produced high amounts of intracellular acetate [78]. Genetically modified *Schizochytrium* mutants overexpressing ACS can therefore readily convert the available pool of acetate to acetyl-CoA, resulting in increased FA and biomass accumulation [78] (Table 1). Thus, utilizing acetate as the major carbon source for acetyl-CoA can occur only when exogenous acetate is supplied to microalgae for heterotrophic growth and FA production [79]. Nevertheless, ACS has been identified in the proteomes of lipid droplets in *C. reinhardtii* [80, 81] and *Dunaliella bardawil* [82], suggesting a possible role of ACS in providing acetyl-CoA at the early stages of FA synthesis. Indeed, upregulation of ACS has been demonstrated recently to provide an alternative route for acetyl-CoA production in oleaginous *C. desiccata*, bypassing the traditional pathway catalyzed by plastidial pyruvate dehydrogenase (PDH), resulting in a coordinated large increase in acetyl-CoA levels that precedes TAG accumulation [83].

#### ATP:citrate lyase

**Fig. 1:**
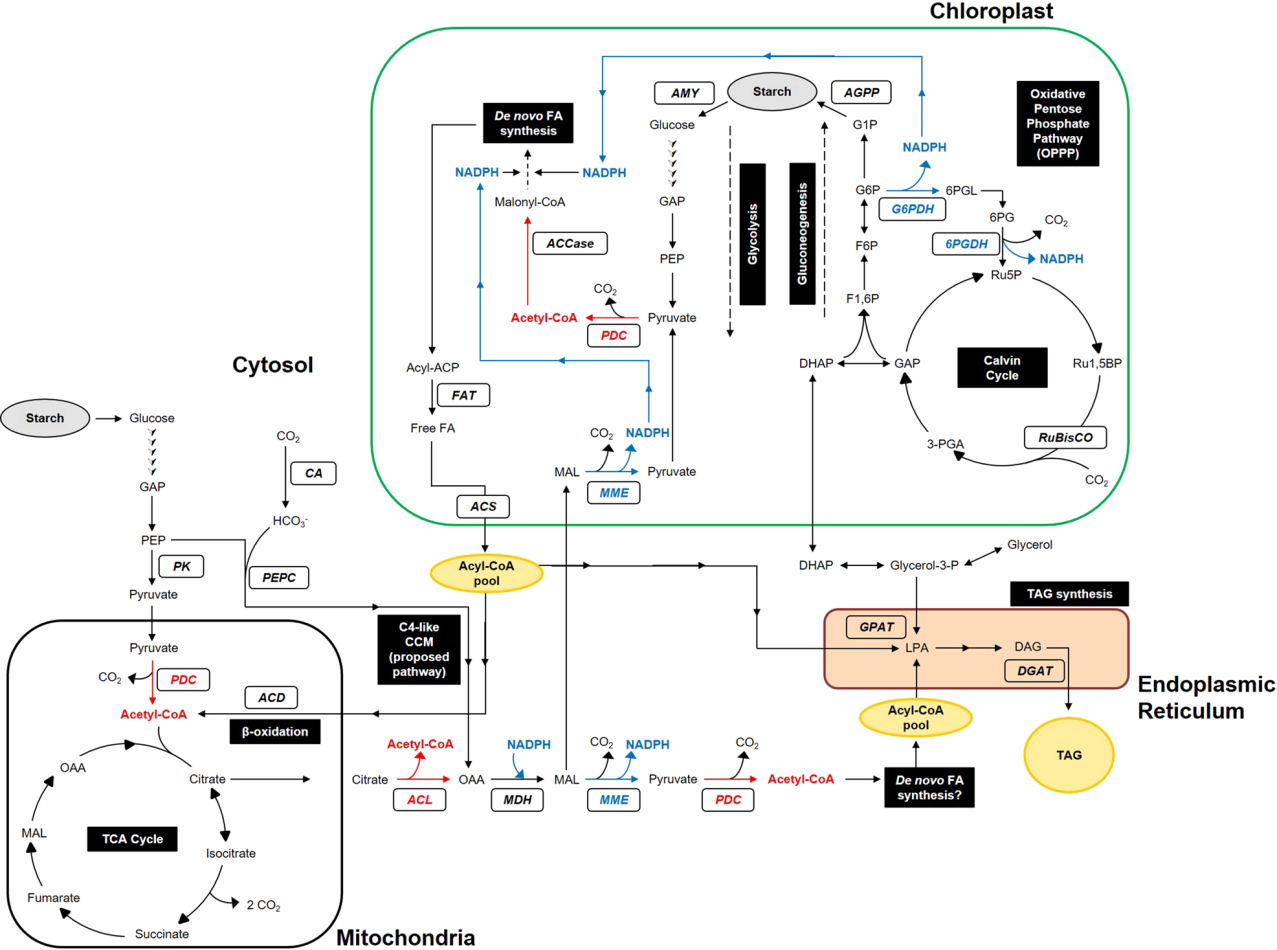
Simplified scheme of central carbon metabolism in microalgae. *Arrows* represent potential carbon fluxes. Enzymes are in *bold* italics. *Blue arrows* represent reducing power (NADPH). *Red arrows* represent acetyl-CoA. *Black boxes* denote pathway names. Neutral lipid droplets found in microalgae consist mostly of triacylglycerols (TAGs), formed by combining FAs and glycerol. *ACCase* acetyl-CoA carboxylase; *ACD* acyl-CoA dehydrogenase; *ACL* ATP-citrate lyase; *ACS* acyl-CoA synthetase; *AGPP* ADP-glucose pyrophosphorylase; *AMY* amylase; *CA* carbonic anhydrase; *DGAT* diacylglycerol acyltransferase; *DHAP* dihydroxyacetone phosphate; *F1,6P* fructose 1,6-bisphosphate; *F6P* fructose 6-phosphate; *FAT* fatty acyl–acyl carrier protein (ACP) thioesterase; *G1P* glucose 1-phosphate; *G6P* glucose 6-phosphate; *G6PDH* G6P dehydrogenase; *GAP* glyceraldehyde 3-phosphate; *GPAT* glycerol-3-phosphate acyltransferase; *MAL* malate; *MDH* malate dehydrogenase; *MME* NADP-malic enzyme; *OAA* oxaloacetate; *PDC* pyruvate dehydrogenase complex; *PEP* phosphoenolpyruvate; *PEPC* PEP carboxylase; *PK* pyruvate kinase; *Ru5P* ribulose 5-phosphate; *Ru1,5BP* ribulose 1,5-bisphosphate; *RuBisCO* Ru1,5BP carboxylase/oxygenase; *3-PGA* 3-phosphoglycerate; *6PGDH* 6-phosphogluconate dehydrogenase

Acetyl-CoA can also be produced from sugars via citrate through ACL which cleaves cytosolic citrate to form acetyl-CoA and oxaloacetate (Fig. 1). Compared to acetate, citrate is produced continuously from the TCA cycle, thus representing a more sustained source of acetyl-CoA for FA synthesis. ACL has often been proposed to be a major rate-limiting enzyme in oleaginous heterotrophs [70, 84], and overexpression of ACL and ME in the yeast *Yarrowia lipolytica* reportedly resulted in a 60-fold increase in lipid yields by providing substrates for the induction of FA synthesis [85] (Table 1). In plants, ACL activity is correlated with lipid accumulation [86] and its mRNA levels coincide with peak cytosolic ACCase expression [87]. In addition, negative regulation of ACL subunit A (ACLA) reduced cuticular wax synthesis in epidermal cells of *Arabidopsis* [88], leading to the notion that increased acetyl-CoA supply in the cytosol may be to support the synthesis and elongation of long-chain FAs. Two species of microalgae, *Nannochloropsis salina* and *Chlorella sp.*, were found to exhibit ACL activity comparable to those in oleaginous heterotrophs [89], indicating that microalgae possess ACL required to utilize citrate as a carbon source for generating acetyl-CoA. Gene expression of cytoplasmic ACL was also upregulated prior to TAG accumulation in *C. desiccata*, but not in low TAG accumulators such as *D. tertiolecta* and *C. reinhardtii* [83]. However, ACL activity would require an efflux of citrate from the mitochondria to the cytoplasm, effectively draining the TCA cycle of its intermediates. The contribution of cytoplasmic acetyl-CoA to FA synthesis in oleaginous microalgae also requires further validation [83]. As mitochondria functions in the light to export citrate via the citrate-oxaloacetate shuttle [90], the exported citrate could thus be exploited to produce acetyl-CoA. However, unlike yeasts where acetyl-CoA can be used directly for fatty acid synthesis in the cytosol [91], microalgae may require the import of carbon substrates into the chloroplast where FA synthesis takes place. Targeting ACL to the plastids of tobacco leaves was previously found to increase fatty acid content by 16% [92], but as the source of plastidial citrate was not identified, it is doubtful that ACL can provide acetyl-CoA for FA synthesis in the chloroplast. Subsequent biochemical and bioinformatics studies in *Arabidopsis* did not reveal the presence of ACL in chloroplasts [87, 93]. Hence, its cytosolic nature means that the supply of acetyl-CoA by ACL is likely to be restricted to FA synthesis within the cytosol.

#### Pyruvate dehydrogenase

Since FA synthesis occurs in the chloroplast, the most efficient way for direct acetyl-CoA synthesis is in the chloroplast itself. Plastidial FA synthesis which relies on acetyl-CoA from ACS or ACL usually applies only to energy-starved or non-green plastids which lack the ability to generate acetyl-CoA locally in the chloroplast [94, 95]. These plastids therefore require the import of intermediate metabolites such as acetate, phosphoenolpyruvate, or pyruvate in order to support the synthesis of acetyl-CoA [95]. In contrast, chloroplasts are capable of independently generating ATP by photosynthesis, which drives the synthesis of energy-rich metabolites such as G3P. Hence, for the majority of photoautotrophic microalgae used to produce renewable biofuels by sequestration of CO_2_, the most common way to produce acetyl-CoA in the plastids is from pyruvate via PDC. The concept that plastidial acetyl-CoA may be derived from pyruvate is substantiated with the isolation of cDNA encoding for subunits of plastidial PDC [96], and organelle fractionation studies demonstrating PDC activity in the plastids [97]. In addition, carbon labeling experiments demonstrated that ^14^C-pyruvate was a superior substrate to ^14^C-acetate for the formation of FAs [97], implying that PDC is sufficient for producing acetyl-CoA for FA synthesis. While mRNA levels of ACS did not correlate with lipid formation in developing seeds of *Arabidopsis*, the mRNA levels of plastidial PDC E1β subunit displayed temporal and spatial accumulation patterns that are consistent with a predominant role for plastidial PDC in FA synthesis [75]. A compelling recent study comparing the transcriptomic and metabolic profiles of oil and date palms reaffirmed the importance of plastid carbon precursor supply for FA synthesis by demonstrating a 24-fold increase in the abundance of plastidial PDH during fruit ripening [65] which suggests an indispensable role of plastidial PDH in storage lipid production.

Similarly in microalgae, the most upregulated genes in the transcriptome under nitrogen depletion were consistently associated with nitrate uptake and assimilation, the PDC, and the TCA cycle [98], with multiple studies reporting an upregulation of plastidial PDC correlating with an increase in overall lipid production [47, 99]. Interestingly, transcript levels of plastidial PDC were two to sixfold higher than those of mitochondrial PDC, supporting the concept that plastid is the primary site of pyruvate conversion to acetyl-CoA [47]. Gene expression of the plastidial PDC E1α subunit was rapidly induced during nitrogen depletion in the high TAG-accumulating microalga *C. desiccata*, in concert with increasing levels of acetyl-CoA [69]. This trend was not observed in two other microalgae species, *C. reinhardtii* and *D. tertiolecta*, which produced significantly lower amounts of acetyl-CoA and did not experience an increase in PDC E1α transcript levels [69]. Additionally, downregulation of plastidial PDC E1α impaired TAG accumulation and photosynthetic activity in nitrogen-depleted *C. reinhardtii* [100], signifying the importance of PDC in supplying carbon precursors for *de novo* lipid synthesis in microalgae (Table 1). However, the negative effects of its downregulation were not so apparent when acetate is present in the media, suggesting that ACS might have a role in PDH bypass, incorporating exogenously available acetate into acetyl-CoA [83]. Furthermore, antisense knockdown of a putative PDC kinase—which deactivates PDC—increased neutral lipid content up to 82% [101] (Table 1). Even under non-nitrogen-limiting conditions, PDC subunits were also observed to be upregulated following transition from starch-rich heterotrophy (dark conditions) to lipid-rich photoautotrophy (light conditions) in the oleaginous *C. pyrenoidosa* [72]. Under elevated doses of CO_2_ which improves lipid accumulation in microalgae, genes involved in carbohydrate metabolic pathways, such as the components of the PDC, were found to be upregulated in *Chlorella sorokiniana*, despite a downregulation of most FA synthesis genes [102]. This implies that the positive correlation between PDC expression and lipid buildup is not primarily confined to nutrient-depleted conditions. The notion is that PDC transforms pyruvate into acetyl-CoA, which may then be used for FA synthesis or channeled to the TCA cycle for cellular respiration.

The generation of plastidial pyruvate is governed by three reactions: by plastidial pyruvate kinase, by pyruvate import into plastids, or by plastidial ME [103]. The glycolytic enzymes phosphoglycerate mutase, enolase, and pyruvate kinase, which produce pyruvate from glycolysis, are notably absent from algal chloroplasts [104], meaning that sources of plastidial pyruvate are likely to be imported into the chloroplast [55, 56] or partially compensated for by plastidial ME [103]. Although pyruvate is relatively small and able to passively permeate lipid bilayers, it is also possible that pyruvate is transported by specialized pyruvate transporters, as evidenced in C4 plants [105] (Fig. 1).

#### Glucose-6-phosphate dehydrogenase

In addition to carbon supply from acetyl-CoA, production of FAs requires the provision of reducing power in the form of NADPH. Generation of NADPH for FA synthesis was mainly attributed to the metabolism of glucose-6-phosphate (G6P), pyruvate, and malate as these metabolites were found to support high rates of FA synthesis in plant plastids [106, 107]. Multiple routes provide NADPH for plastids, including plastidial glycolysis, photochemical reactions, import of metabolites from cytosol or mitochondria, or the Oxidative Pentose Phosphate Pathway (OPPP) [108]. The OPPP in particular, was found to be enhanced along with the Calvin cycle when algal cells were exposed to higher CO_2_ concentrations; their respective enzyme activities and gene expression increased in parallel with photosynthetic performance, growth rate, and lipid productivity [109].

G6P can be utilized in two pathways: Glycolysis and the OPPP; the latter is a major source of reducing power for biosynthetic processes. Multiple studies show that plastidial G6P flux through the OPPP stimulates the incorporation of carbon from pyruvate into FAs [97, 107, 110], suggesting that there is an interaction between provision of carbon substrates and reducing power to simultaneously increase FA synthesis. The first committed reaction of the OPPP is catalyzed by G6PDH, which metabolizes G6P to produce NADPH [111]. G6PDH is considered to be an important source of NADPH in heterotrophic plant plastids producing high amounts of FAs, but which are not able to synthesize NADPH by photosynthesis [95, 112]. Nevertheless, there is conflicting evidence regarding the dependence on OPPP to sufficiently meet the demands for FA synthesis. *Brassica napus* and maize embryos demonstrated 10 and 36% of carbon influx to OPPP, respectively, accounting for only 22% of NADPH required by FA synthesis [113, 114]. In sunflower embryos, however, NADPH production by OPPP was more than enough to supply for FA synthesis [115]. Metabolic flux analysis for the microalga *C. pyrenoidosa* found that culture conditions determine glycolytic flux through the OPPP [116]. When in an autotrophic state, glucose flux through the OPPP was very small as the synthesis of NADPH is provided by photosynthetic electron transport, but during heterotrophic growth, 90% of glucose proceeds via G6PDH [116]. Recent reports on *C. protothecoides* adds further weight to this notion by demonstrating that under nitrogen-limited conditions, [13-C] glucose flux to glycolysis through the OPPP increased from 3 to 20% of the glucose uptake, reflecting increased NADPH requirements for lipid synthesis [71, 117]. On the other hand, halotolerant microalgae such as *Dunaliella salina* may exhibit higher OPPP flux and increased G6PDH proteins under autotrophic growth as the OPPP appears to play a major role in osmoregulation [118, 119]. When the oleaginous microalgae *N. oleoabundans* was subjected to nitrogen depletion inducing the accumulation of lipids, it displayed an upregulation of genes encoding for the OPPP including G6PDH, suggesting that the OPPP could be activated as a response to supply reductants for FA synthesis and/or inorganic nitrogen assimilation [25]. In *P. tricornutum*, high activity and mRNA expression of G6PDH were observed as cells were exposed to increasing levels of CO_2_ [109], suggesting that NADPH produced through OPPP might play an important role in both growth and lipid synthesis under higher CO_2_ concentration. Most intriguingly, recent efforts to identify a critical determinant of FA synthesis have pointed to the OPPP as the NADPH producer responding to lipogenesis [66]. While studying global gene expression patterns, Chen et al. found that oleaginous microbes such as *C. reinhardtii* and *Mucor circinelloides* showed upregulated expression of 6-phosphogluconate dehydrogenase (6-PGDH) and G6PDH under nitrogen starvation, but they remain unchanged in the non-oleaginous fungi *Aspergillus nidulans*. G6PDH was identified to be particularly important for FA synthesis as RNAi knock down of G6PDH resulted in a corresponding 40% decrease in both NADPH levels and FA content, while suppression of 6-PGDH and ME had a much lesser effect on NADPH and FA accumulation. G6PDH, and by extension the OPPP, could therefore be an indispensable source of NADPH which enables efficient FA synthesis in oleaginous microbes.

#### NADP-malic enzyme

That NADPH produced from OPPP only partially fulfills the demand for reducing power by FA synthesis [113, 114] indicates that other intraplastidial sources of reducing power may be available to compensate for the need for reductants [120, 121]. Malate, when supplied alone, was able to support the highest rates of FA synthesis in plastids [107, 122]. In contrast, G6P and pyruvate must be supplied together to obtain rates of FA synthesis comparable to those supported by malate [107]. These observations reveal another potential source of reductants for FA synthesis—utilization of malate by plastidial NADP-dependent malic enzyme (ME). ME produces NADPH by catalyzing the decarboxylation of malate to pyruvate, while PDC subsequently converts pyruvate to acetyl-CoA, thus providing for the two key substrates required for FA synthesis. Import of malate into the plastids relies on the translocation of mitochondrial malate or the refixation of CO_2_ into oxaloacetate (OAA) in the cytosol by phosphoenolpyruvate carboxylase, followed by conversion of OAA into malate catalyzed by malate dehydrogenase [121, 123] (Fig. 1). The latter route involves a proposed C4 carbon-concentrating mechanism in microalgae which describes a malate/pyruvate shuttle permitting cytosolic malate into the chloroplast where plastidial ME releases CO_2_ and NADPH [64, 123]. Mitochondrial TCA cycle reactions were found to be accelerated under N-depleted conditions producing OAA and/or malate [47, 124], suggesting that these metabolites may be crucial to support increased FA synthesis. Malate can be transported to the chloroplast via malate transporters [125] and utilized by plastidial ME producing pyruvate and NADPH, which flows into FA synthesis. Moreover, the CO_2_ produced by this reaction could be directly fixed by RuBisCO to increase photosynthetic capacity. This repartitioning route for supplying carbon precursors was supported by a computational hypothesis testing approach, where the authors assign to the TCA cycle a central role for de novo FA synthesis by providing excess malate [124].

Recent studies have provided evidence to establish the possible role of ME in microalgal lipid synthesis. Expression levels of ME were correlated with an increase in lipid content during nitrogen limitation [64, 126]. When sesamol, a known inhibitor of ME, is added to nitrogen-limited cultures of *Nannochloropsis* sp. and *H. pluvialis*, it reduced both growth and fatty acid accumulation [127], suggesting that ME might be important for providing NADPH for essential cellular functions as well as for FA synthesis. Indeed, overexpression of ME in *P. tricornutum* enhanced neutral lipids by 2.5-fold, while maintaining similar growth rates to the wild type [128] thus enabling the increased production of lipids without sacrificing biomass. Introduction of heterologous *P. tricornutum* ME into *C. pyrenoidosa* also increased neutral lipids up to 3.2-fold relative to wild type, with the mutants achieving lipid contents of between 30 and 40% relative to wild-type strains which exhibited 12.7% lipids [129] (Table 1). However, low ME activity was also detected in some microalgae species [89, 117] which may limit NADPH supply, suggesting that ME could be rate limiting in the provision of substrates for FA production. Several isoforms of ME exist with distinct localization in the mitochondria, cytosol, and plastid [130, 131], possibly carrying out different functions. In oleaginous heterotrophic species, cytosolic ME is proposed to form a lipogenic metabolon with ACL and ACCase, where it supports FA synthesis by continuous NADP reduction [84]. Overexpression of mitochondrial ME did not result in any significant changes in lipid content [132], while the use of cytosolic ME may only be effective when fatty acid synthesis occurs in the cytosol [133, 134]. Plastidial ME, on the other hand, was found to increase over sevenfold in transcript levels during nitrogen depletion in *P. tricornutum* [64], suggesting that it could provide for essential reducing power for FA synthesis in photoautotrophic cells. In the model microalga *C. reinhardtii*, six isoforms of ME exist, with two predicted plastidial isoforms, but they are associated with anaerobic metabolism and not with FA synthesis [104, 135]. Hence, although ME has the potential to supply reductants for increased FA synthesis, like G6PDH and the OPPP, its role within specific cell types relating to lipid accumulation has to be pre-determined.

#### Multi-gene approach

Individual genes participating in the supply of acetyl-CoA or NADPH are claimed by various studies to be rate limiting for FA synthesis [37, 70, 133]. However, it is likely that a multi-gene approach may be required to completely rewire an organism’s ability to switch from non-oleaginous to oleaginous, without compromising growth. For example, the claim that NADPH provision by ME was a rate-limiting step in FA synthesis was previously backed by observations that ME overexpression in the fungus *M. circinelloides* led to a 2.5-fold increase in FAs [133]. This conclusion was challenged by another report revealing that it is actually leucine auxotrophy—which the previous authors used for selection—that decreased FA content by 2.5-fold, and that ME overexpression did not translate into higher lipid levels despite an increase in ME activity [136]. It was thus proposed that the leucine metabolism pathway, by participating in the generation of acetyl-CoA substrates, may be critical for FA synthesis in *M. circinelloides*. A subsequent study showed that ME overexpression increased FA content by only 30% relative to control, despite a twofold increase in ME activity, suggesting that ME may not be the sole rate-limiting enzyme, but does play a role during FA synthesis in oleaginous fungi [137]. Recently, a large-scale genomic engineering of *Y. lipolytica* involved the simultaneous overexpression of ACL and ME to increase acetyl-CoA and NADPH supply, while DGAT was also overexpressed to increase carbon flux towards TAG synthesis [85]. In addition, these targets were multiplexed with deletions of genes which serve to reduce FA catabolism by inhibiting the peroxisomal β-oxidation pathway. Consequently, the cells became lipid saturated up to 90% biomass with lipid titres exceeding 25 g/L, which is a 60-fold improvement over the parental strain [85]. Thus, increasing of FA synthesis in microorganisms may not simply be explained by the overexpression of a single enzyme, but influenced by a combination of pathways spanning FA synthesis, TAG synthesis, and central carbon metabolism, including production of FA precursors like acetyl-CoA and NADPH.

## Conclusions

Although considered to be a promising replacement to plants as biofuel feedstocks, studies have established that the economics of producing biofuels from microalgae needs to be significantly improved. The general consensus highlights a need for higher lipid and biomass production rates, as well as lowering of production costs, primarily through efficient harvesting of biofuels. The regulatory and metabolic networks of microalgae during stressed and non-stressed conditions are vastly different. Under normal non-stress conditions, microalgae are genetically programmed to synthesize and store starch to support growth and cell division, as they are evolved to be highly specialized for carbon capture and carbohydrate synthesis. On the other hand, microalgae subjected to stress conditions tend to accumulate high amounts of storage lipids at the expense of growth. As cell growth and lipid accumulation are usually mutually exclusive for photosynthetic microalgae, a key goal is to devise strategies to divert the flow of photosynthetic carbon towards lipid synthesis.

The dilemma between stimulating lipid production while maintaining biomass productivity has been claimed by some recent reports to be solved by the targeting of candidate genes and pathways, but the lack of an efficient and reproducible technique for transforming non-model microalgae is proving to be a major stumbling block in approaches to genetically engineer superior strains with high lipid- and biomass-accumulating potential. If demonstrated to be reliable, genetic tools such as those of homologous recombination may prove to be the breakthrough needed for targeted mutagenesis in microalgae. Applying available knowledge with novel genetic methods holds great promise in unlocking the potential of photosynthetic microalgae as an important source of renewable biofuel that will not compete for arable land, freshwater, or food production. While genetically engineered microalgae hold great promise for commercial production of fuels and feedstocks, they also pose possible risks to the health of ecosystems if they escape from open ponds. For example, contamination by a genetically engineered microalgae that are resistant to viruses may give it a competitive advantage to growth in the local environment, allowing for dense growth without dying which results in devastating effects on the existing fauna. Thus, before novel mutant strains are introduced as feedstocks for industrial use, key biosafety issues should be addressed by formal environmental risk assessments to properly guide the sustainable development of microalgal biofuels.

## Abbreviations

ACCase: acetyl-CoA carboxylas
ACL: ATP:citrate lyase
ACS: acyl-CoA synthetase
FA: fatty acid
G6PDH: glucose-6-phosphate dehydrogenase
ME: NADP-malic enzyme
PDC: pyruvate dehydrogenase complex
TAG: triacylglycerol
